# Sanders type IIIAB calcaneal fracture without broken lateral wall: A case report and review of literature

**DOI:** 10.1097/MD.0000000000009926

**Published:** 2018-02-16

**Authors:** Zhenshuang Yue, Yanghua Tang, Zhongqing Hu, Wenjie Zheng

**Affiliations:** Department of Orthopedics, Xiaoshan Traditional Chinese Medical Hospital, Hangzhou, Zhejiang, P.R. China.

**Keywords:** calcaneal fracture, CT, lateral wall

## Abstract

**Rationale:**

The calcaneal fracture without broken lateral wall is rare and the open anatomic reduction and internal fixation (ORIF) is necessary when the subtalar joint articular surface is broken and collapsed.

**Patient concerns:**

A 45-year-old male was admitted to our department with complaints of heel pain and swelling after falling down from 1-m-high stone. He was unable to bear weight on his right foot.

**Diagnoses:**

Imaging studies confirmed that it was a sanders type IIIAB calcaneal fracture without broken lateral wall and the middle part of the posterior calcaneal articular facet collapsed.

**Interventions:**

ORIF of intraarticular calcaneal fracture with the locking calcaneal plate was performed.

**Outcomes:**

The patient recovered completely after 16 weeks and was able to participate in his usual work.

**Lessons:**

Based on this case and literature we reviewed, computed tomography scan (CT scan) should be used to diagnose and evaluate the severity of calcaneal fractures. Currently, ORIF was the preferred surgical treatment option when dealing with displaced intraarticular calcaneal fractures.

## 1 Introduction

Calcaneal fractures are the most commonly fractured tarsal bones and account for approximately 60% of all tarsal fractures.^[1]^ Approximately, 75% of calcaneal fractures are intraarticular fractures in nature.^[2–4]^ In clinical cases, the main pathological features of calcaneal fractures are collapse of subtalar joint articular surface, deformity of calcaneal body, and destruction of lateral wall. However, the case with sanders type IIIAB calcaneal fracture without broken lateral wall has not been reported before. Here, we reported a rare case with sanders type IIIAB calcaneal fracture of the right foot.

## 2 Methods

The private information and medical records were obtained from patient with informed consent and with approval of the institutional ethics committee.

## 3 Case report

A 45-year-old male was admitted to our Foot and Ankle Surgery Department, with a complaint of right foot swelling and pain. The patient fell from 1 m high and landed heavily on his right heel.

Physical examination revealed palpable swelling and ecchymosis around the entire foot and ankle. No obvious sense of bone rubbing was shown. Motion of the toes could not cause pain. The flexion–extension movement of the ankle joint was limited due to the swelling and pain. In addition, the patient denied toes paresthesia and the dorsalis pedis artery pulse was normal.

**Figure 1:**
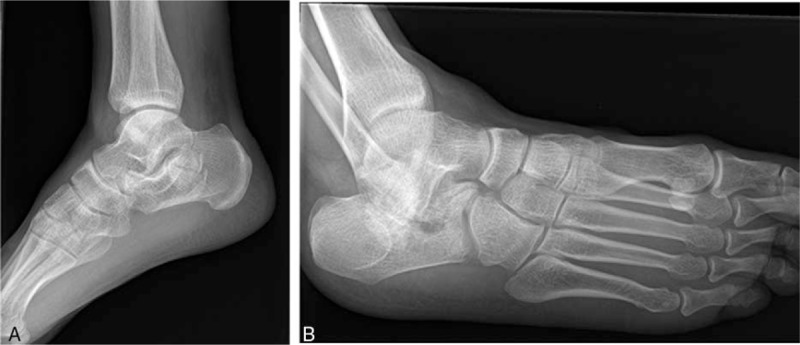
(A) The X-ray showed bony irregularity and a higher density region on the corpus calcaneus. (B) The bone defect was found in the forepart of the calcaneus by oblique radiograph.

**Figure 2:**
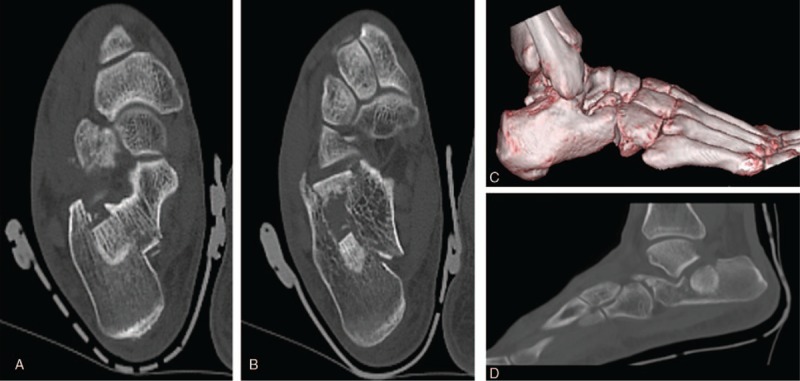
(D) CT scan showed collapsed calcaneal fracture in the middle of posterior facet. (A–C) The lateral wall of calcaneus was intact.

The X-ray showed bony irregularity and a higher density region on the corpus calcaneus (Fig. 1A). The bone defect was found in the forepart of the calcaneus by oblique radiograph (Fig. 1B). Computed tomography (CT) scan showed collapsed calcaneal fracture in the middle of posterior facet (Fig. 2D). Surprisingly, the lateral wall of calcaneus was intact (Fig. 2A–C).

**Figure 3:**
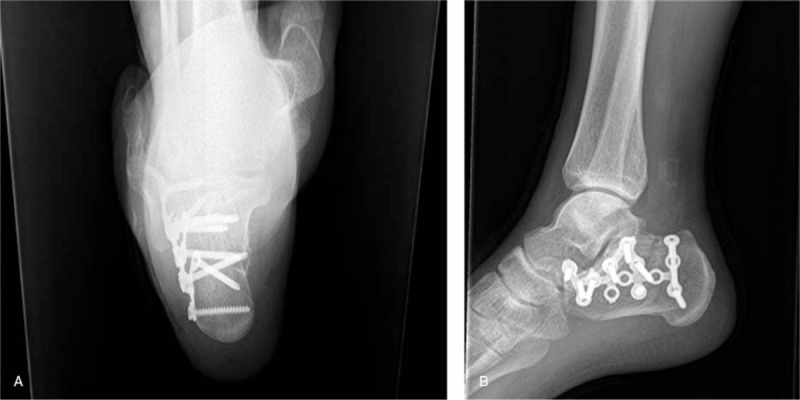
(A and B) The fracture was reducted and fixed properly.

The patient underwent open anatomic reduction and internal fixation (ORIF) for articular fracture of the calcaneus by standard “L” incision on the 9th day after injury. We confirmed that the lateral calcaneal wall was intact during operation. Therefore, the “V” osteotomy of lateral wall was performed in the operation. Besides, we also found that the fragments mainly located in the central part of the posterior calcaneal facet and the calcaneal body was slightly introversive and shortened. At last, the fracture was reducted and fixed properly (Fig. 3A and B).

Rehabilitation included active and passive range of motion exercises of the ankle and subtalar joint mobilized from the second postoperative day. The patient was restricted to be kept nonweight-bearing for 8 weeks until X-ray showed good fracture heeling. In the 4th month, he was participating in usual work and completely pain free.

## 4 Discussion

The injury mechanism of calcaneal fractures is still unclear. However, most of the calcaneal fractures are attributed to shearing stress adjoined with compressive forces combined with a rotary direction.^[5]^ There are many classifications based on different theories and different emphasis to better understand the calcaneal fractures.^[6–9]^

The Sanders classification system is the most commonly used system for categorizing intraarticular fractures.^[10,11]^ This case is type IIIAB according to Sanders classification system. It has never been reported in the literatures about Sanders type IIIAB calcaneal fractures without broken lateral wall. The imaging examinations often suggest that 2 fracture lines are present,^[12]^ 1 lateral and 1 central, the posterior calcaneal facet is collapsed and compressive, the calcaneal body is introversive and shortened in Sanders type IIIAB. We can notice “crescent sign” in lateral radiograph as a result of impaction of articular fragment into the body of the calcaneus. Therefore, the patients with Sanders type IIIAB calcaneal fractures should be treated with ORIF to correct the deformities. However, Sanders type IIIAB calcaneal fractures with intact lateral wall are clinically rare. Our patient could not describe the process of injury, especially the landing surface and the person's posture. Therefore, further studies are required to truly know the injury mechanism of this fracture.

In our case, the calcaneal fracture was mainly evaluated by CT scan. The patient recovered completely after 16 weeks and was able to participate in his usual work.
